# Low Molecular Weight Bio-Polyamide 11 Composites Reinforced with Flax and Intraply Flax/Basalt Hybrid Fabrics for Eco-Friendlier Transportation Components

**DOI:** 10.3390/polym14225053

**Published:** 2022-11-21

**Authors:** Claudia Sergi, Libera Vitiello, Patrick Dang, Pietro Russo, Jacopo Tirillò, Fabrizio Sarasini

**Affiliations:** 1 Department of Chemical Engineering Materials Environment, Sapienza Università di Roma and UdR INSTM, 00184 Rome, Italy; 2 Department of Chemical, Materials and Production Engineering, University of Naples Federico II, 80125 Naples, Italy; 3 Arkema High Performance Polymers, Research & Development, Cerdato, 27470 Serquigny, France; 4 Institute for Polymers, Composites and Biomaterials—National Council of Research, 80078 Pozzuoli, Italy

**Keywords:** polyamide 11, basalt, flax, hybrid composites, low-velocity impact, temperature, polymer matrix composites

## Abstract

The transportation sector is striving to meet the more severe European legislation which encourages all industrial fields to embrace more eco-friendly policies by exploiting constituents from renewable resources. In this framework, the present work assessed the potential of a bio-based, low molecular weight PA11 matrix reinforced with flax and intraply flax/basalt hybrid fabrics. To this aim, both quasi-static and impact performance were addressed through three-point bending and low-velocity impact tests, respectively. For hybrid composites, the effect of stacking sequence, i.e., [0/0] and [0/90], and fiber orientation were considered, while the effect of temperature, i.e., −40 °C, room temperature and +45 °C, was investigated for laminates’ impact response. The mechanical experimental campaign was supported by thermal and morphological analyses. The results disclosed an improved processability of the low molecular weight PA11, which ensured a manufacturing temperature of 200 °C, which is fundamental to minimize flax fibers’ thermal degradation. Both quasi-static and impact properties demonstrated that hybridization is a good solution for obtaining good mechanical properties while preserving laminates’ lightness and biodegradability. The [0/90] configuration proved to be the best solution, providing satisfying flexural performance, with an increase between 62% and 83% in stiffness and between 19.6% and 37.6% in strength compared to flax-based laminates, and the best impact performance, with a reduction in permanent indentation and back crack extent.

## 1. Introduction

Most industrial sectors are making efforts to design and produce components with a lower carbon footprint and a lower environmental impact to redress the strong environmental pollution and to face the ever-increasing waste disposal dilemma [1]. Greener policies intended to boost the use of biodegradable and bio-based materials, to reduce wastes pressure on landfills and to promote a circular economy perspective focused on materials reuse and recycle [2,3] were forced by the more restrictive European legislations. Among them, the 2000/53/EC regulation was specifically addressed to vehicles’ end-of-life and presented a big challenge for the automotive sector, which makes a massive use of polymers and fiber-reinforced polymers (FRP) to ensure high mechanical-property standards while reducing vehicles’ weight and the related fuel consumption. In particular, 8.8% of the 55 million tons of plastics destined to Europe in 2020 were meant to cover the automotive industry demand [4].

Considering that the automotive field covers almost 40% of the global polyamide (PA) demand [5], the exploitation of natural resources to synthesize eco-sustainable PA is a strategic way to face the environmental pollution challenge from the polymeric matrix perspective. For example, the bio-PA11 by Arkema is completely synthesized from renewable resources, i.e., castor oil [6], allowing for a reduction in environmental impact while preserving mechanical reliability. Indeed, PA11 displays only a slight decrease in the mechanical performance compared to the fully petroleum-based PA12, while ensuring a reduction of CO_2_ emissions from 6.9 kgCO_2eq_/kg to 4.2 kgCO_2eq_/kg [7].

If polymers’ carbon footprint reduction is a first valid initiative to meet the European legislation requirements, further efforts are necessary to solve the issue even from the fibrous-reinforcement perspective. According to the data reported by AVK for 2021, glass FRPs still account for more than 95% of the overall market [8]. A first attempt to assess the problem was made with vegetable fibers to obtain fully bio-based and partially/totally biodegradable composites [9]. On these heels, many European and North American companies, i.e., BASF, Rieter Automotive, Delphi Interior Systems and Visteon Automotive, adopted this strategy [10,11].

Undoubtedly, vegetable fibers offer many advantages throughout the whole life cycle of the component, i.e., production, in-service and disposal. Their renewable origin grants a significant reduction in the production process emissions. For example, the production of hemp and China reed fibers ensures a reduction of almost 97% and 87% in the CO_2_ and SO_x_ emissions with respect to glass fibers, respectively [12,13]. Their low cost, low density and high specific stiffness allow for the reduction of vehicles’ fuel consumption and greenhouse gas emissions, providing a reduction of 30% in components’ weight and of 20% in their cost [14]. Finally, their biodegradability ensures an easier disposal at the end of the components’ life cycle.

Despite all these benefits, vegetable fibers struggle to become competitive on the market, being unable to ensure the same mechanical performance achievable with glass fibers due to the inherently higher mechanical properties of glass and to vegetable fibers’ hydrophilicity, which counteracts polymeric matrixes’ hydrophobicity, generating a weak interfacial adhesion [15]. The low thermal stability is another drawback which delays vegetable fibers’ spread in the industrial landscape [16]. Indeed, temperature lower than 200 °C must be used to avoid fiber degradation, thus decreasing the number of polymers exploitable and their processability.

All these limitations and the rush for bio-based solutions pushed to find another natural solution which was found in the basalt mineral volcanic rock. It proved to be a perfect solution to obtain mechanical properties’ competitiveness with glass ones, a greener manufacturing process where no additional chemicals are required and energy consumption is significantly reduced [17], and an easier recycling and recovery of the fibers at the end of composite life cycle [18].

Notwithstanding all the advantages, basalt fibers are not biodegradable and display a density comparable with glass ones, thus losing all the advantages acquired with vegetable reinforcements. Hybridization is a feasible strategy to produce FRPs characterized by satisfying mechanical properties, a lower weight and cost, an improved eco-friendliness and a partial biodegradability. Hybridization benefits are undeniable, as confirmed by the huge number of works that applied this approach for the glass/vegetable fibers system [19,20], but further environmental advantages may be gained by replacing glass fibers with basalt. This idea was assessed using various hybrid configurations with different vegetable fibers such as hemp [21,22] and jute [23,24], but among them, the flax/basalt one was the most widely investigated [25,26,27] thanks to flax’s superior mechanical properties among vegetable fibers [28].

Considering the strategies available to increase composites’ eco-friendliness from both the matrix and fiber perspective, the present study aims to assess the potential of a green composite produced with a bio-PA11 matrix reinforced with an intraply flax/basalt hybrid fabric for automotive components. The effect of fibers’ hybridization on a different bio-polyamide was already assessed by Bazan et al. [29], who investigated a bio-polyamide 10.10 reinforced with aramid/basalt fibers, and by Armioun et al. [30], who used a PA11 as a matrix and a wood/carbon combination as a hybrid reinforcement. Some preliminary studies on the hybrid configurations proposed in this work were also performed by Russo et al. [31] and by Sergi et al. [32] from the quasi-static and impact response perspective, respectively, but some important steps forward were taken in this work.

In particular, a low molecular weight bio-PA11 was used as a matrix, allowing for the processing of the composite at lower temperatures, i.e., lower than 200 °C, thus reducing flax fibers’ thermal degradation. Russo et al. [31] and Sergi et al. [32] assessed the effect of a butyl-benzene-sulfonamide plasticizer on the quasi-static and impact properties of the PA11 flax/basalt composite, respectively, but none of them evaluated the effect of the stacking sequence or fiber orientation. Moreover, Sergi et al. evaluated the impact response at room temperature and +80 °C, but no information is available about the cryogenic impact response. In light of this, the proposed study investigated the quas-istatic, i.e., three-point bending, and impact properties of the low molecular weight bio-PA11 reinforced with the intraply flax/basalt hybrid woven fabric assessing the effect of stacking sequence and fiber orientation. In particular, the [0/0]_s_ and the [0/90]_s_ stacking sequences were considered, and a direct comparison with a flax-reinforced PA11 was provided to disclose the significant improvement offered by hybridization. Considering that impact resistance is a key feature for the transportation field, composite impact response was investigated as a function of both impact energy, i.e., 10 J, 20 J and 30 J, and operating temperature, i.e., −40 °C, room temperature and +45 °C. The mechanical characterization was accompanied by a morphological and structural characterization through scanning electron microscopy and X-ray diffraction; by a thermal characterization through thermogravimetric analysis, differential scanning calorimetry and dynamic mechanical analysis; and by a post-impact analysis through profilometry. Moreover, a rheological study aimed at evaluating matrix processability was also carried out.

## 2. Materials and Methods

### 2.1. Materials and Manufacturing Process

The low molecular weight bio-PA11 FMNO Rilsan^®^ by Arkema (Serquigny, France) was selected as a polymer matrix, while a flax and intraply flax/basalt hybrid fabric were selected as reinforcements. In particular, a 2/2 flax twill supplied by Composites Evolution Ltd. (Chesterfield, UK) with an areal weight of 300 g/m^2^ and a 2/2 interwoven flax/basalt hybrid twill provided by Lincore^®^ with an areal density of 360 g/m^2^ and a 50 wt% of basalt and flax fibers were used to produce the laminates.

Composites were manufactured by hot compression molding using a P400E hydraulic press by Collin GmbH (Maitenbeth, Germany) applying the film-stacking technique. In particular, ten plies of reinforcement were alternated with 100 µm-thick PA11 films and were compressed at 200 °C, increasing the applied pressure from 0.5 to 3 MPa with a 0.5 MPa increment every two minutes. Before composites manufacturing, the PA11 matrix was vacuum oven dried at 70 °C overnight. Three different laminate configurations were considered: PA11 reinforced with flax (Flax), PA11 reinforced with the intraply hybrid fabric using a [0/0]_s_ stacking sequence (Hybrid [0/0]) and PA11 reinforced with the intraply hybrid fabric using a [0/90]_s_ stacking sequence (Hybrid [0/90]). The main characteristics of the three configurations investigated are reported in Table 1.

### 2.2. Rheological Characterization and Molecular Weight Assessment

Rheological analyses were performed on the polyamide 11 films provided by Arkema using a stress-controlled rotational rheometer AR-G2 by TA Instruments (New Castle, DE, USA) equipped with 25 mm diameter parallel plates. Consecutive frequency scan tests were carried out on the same sample over a time interval of approximately 16 min, from the frequency ω = 100 up to 1 rad s^−1^ at three temperatures: 200 °C, 210 °C and 220 °C. Before the analyses, PA11 films were dried at T = 70 °C under vacuum overnight (i.e., 12 h). 

To simulate the environment of the compression molding process, the experiments were run in air atmosphere. The complex viscosity (η*) was recorded as a function of frequency in the linear elastic regime (strain amplitude of 5%), in turn identified by preliminary strain amplitude tests.

Size exclusion chromatography (SEC) was performed to check if there was an evolution of the M_n_ or M_w_ during processing of the composites. The samples were dissolved for 24 h at room temperature in hexafluoroisopropanol (HFIP). The molecular weights are given in PMMA equivalent (g/mol).

### 2.3. Thermal and Structural Characterization

Thermal and microstructural characterizations were performed to analyze and interpret material mechanical response. Thermogravimetric analysis (TGA), Differential Scanning Calorimetry (DSC) and Dynamic Mechanical Analysis (DMA) were used to for thermal characterization, while X-ray diffraction (XRD) was employed for structural characterization. Three samples for each characterization technique and each configuration were tested.

TGA was performed on both PA11 neat matrix and the related composites with a Setsys Evolution by Setaram according to ISO 11358. Tests were performed in an inert nitrogen atmosphere from room temperature to 800 °C, employing a heating ramp of 10 °C/min. DSC was carried out on the PA11 matrix with a DSC 214 Polyma by Netzsch (Selb, Germany), according to ISO 11357. Specimens were tested in a nitrogen atmosphere and in a temperature range from 20 °C to 250 °C using a heating/cooling ramp of 10 °C/min. The specimens were subjected to a first heating to remove the thermal history, and the data resulting from the cooling and second heating were used for the analysis. By applying Equation (1), where ΔH*_m_* is the melting enthalpy evaluated from the DSC curve and ΔH*_m_*_0_ is the melting enthalpy of the PA11 matrix fully crystallized (ΔH*_m_*_0_ = 226.4 J/g) [33], it was also possible to evaluate matrix crystallinity X*_c_*:

(1) $$X_{c}=\frac{{\Delta H}_{m}}{{\Delta H}_{m0}}*100$$

DMA was performed on the neat matrix and on all laminates’ configurations along both flax and basalt directions with a DMA 242 E Artemis by Netzsch according to ISO 6721. Samples with a length of 60 mm and a width of 10 mm were tested using a 3-point bending configuration, a temperature range from 20 °C to 100 °C, a heating ramp of 2 °C/min, an amplitude of 30 μm and a frequency of 1 Hz. Finally, XRD analysis was performed with a Philips X’Pert PRO diffractometer on flax and flax/basalt hybrid composites collecting the spectra in the range of 2θ = 10°–40° using a step size of 0.02°, a time per step of 3 s and a CuK_α_ monochromatic radiation (40 kV–40 mA).

### 2.4. Quasi-static Characterization: Flexural Properties 

Flax and flax/basalt PA11 composites were subjected to quasi-static characterization in 3-point bending with a Zwick/Roell Z010 universal testing machine equipped with a 10 kN load cell. Tests were carried out on rectangular specimens with a 16:1 span-to-thickness ratio according to ASTM D790 and a test speed of 2.5 mm/min. For the hybrid composite configurations, tests were performed along both flax and basalt directions to disclose potential variations and anisotropy in composite mechanical behavior.

After mechanical testing, specimens were subjected to fracture surface analysis through an FE-SEM MIRA 3 by Tescan. All specimens were sputter-coated with a thin layer of gold to prevent charging because of their low electrical conductivity. Sputter coating was carried out with an Edwards S150B sputter coater.

### 2.5. Impact Characterization

The impact properties of the materials under study were evaluated through low-velocity impact tests using an instrumented drop weight tower Instron/Ceast 9340. Tests were performed with a hemispherical impactor with an overall mass of 3.055 kg and a diameter of 12.7 mm, using a sample holder with a circular unsupported area of 40 mm in diameter. A pneumatic system assured specimen clamping to sample holder and an anti-rebound system ensured impactor block after the rebound to prevent a second impact. Operating temperature effect was evaluated performing tests at room temperature (25 °C), −40 °C and +45 °C after conditioning the specimens for two hours to ensure a homogeneous temperature profile throughout the material. The combinations of temperature and impact energies investigated for each composite configuration are reported in Table 2.

Impacted specimens were also subjected to a post-impact analysis to evaluate damage extent as a function of impact energy and operating temperature. The residual indentation depth was assessed through profilometry using a laser profilometer Talyscan 150 by Taylor Hobson and a scan speed of 8500 μm/s. The resulting scanned surface was analyzed with the software TalyMap 3D. The back crack extent was also quantified with the image processing software Image J by analyzing the impacted specimen photographs. 

## 3. Results and Discussion

### 3.1. Rheological Characterization and Molecular Weight Assessment

**Figure 1:**
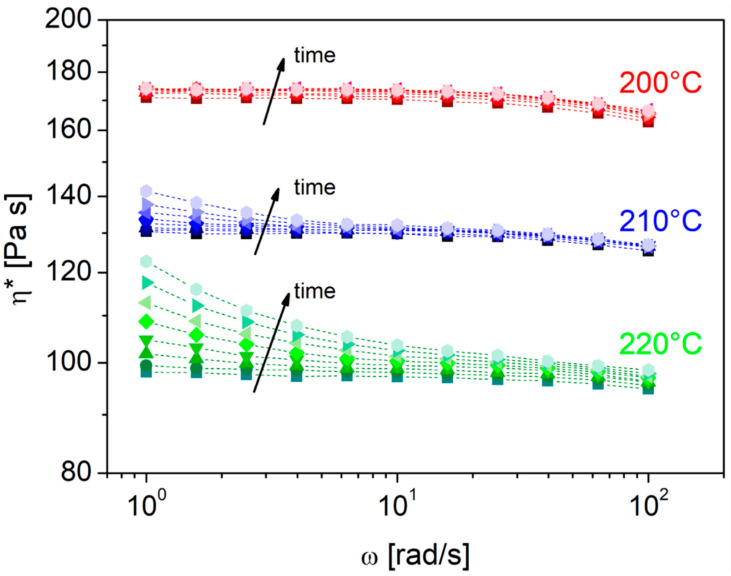
Variation of the complex viscosity (η*) of polyamide 11 in air at three different temperatures: from bottom to top, 220 °C, 210 °C and 200 °C.

The evolution over time of the complex viscosity of the PA11 matrix subjected to consecutive frequency scan tests at three different temperatures is shown in Figure 1. As expected, the higher the test temperature, the lower the viscosity, which, among other things, at the lowest temperature examined (200 °C), shows an almost Newtonian trend apparently not influenced by time. On the contrary, at temperatures of 210 and 220 °C, by repeatedly testing the same sample, an increase in the complex viscosity is evident, with an increasingly marked upward trend of the curves as the test temperature increases: the effect is particularly accentuated in the low-frequency region, in correspondence of which, notoriously, the response of large portions of chains is detected.

This behavior, typical of condensation polymers such as polyamides and polyesters, has been the subject of previous research, according to which the increasing evolution of viscosity and of some viscoelastic parameters is due to the concomitant occurrence of various structural changes. These phenomena are related to both the intrinsically hygroscopic character of these polymers, which inevitably influences the rheological behavior and the optimal process modalities of the same, and the environment in which the material is tested (inert or air).

In general, if the moisture content of the material is less than that of thermodynamic equilibrium, it is reasonable to attribute the increase in viscosity to an increase in the molecular weight of the material due to the occurrence of post-condensation phenomena. In practice, this mechanism is prevalent if we consider the behavior of the melt in an inert environment (nitrogen) [34]. Conversely, in the air, it is very likely that simultaneous thermo-degradative phenomena such as chain scissions and cross-linking occur. It is reasonable to believe that these phenomena begin at the edge of the disk-shaped sample and then propagate over time in the bulk of the sample between the plates thanks to the combination of air and humidity of the material [35]. Filippone et al. [35], combining rheological tests and molecular measurements such as size exclusion chromatography and mass-chromatography, have shown that the increase in complex viscosity over time is essentially related to the extension of cross-linked polymer fractions (insoluble gels) and, therefore, the hindering of the macroscopic flows.

In light of the previous considerations, to limit the occurrence of the aforementioned structural changes and avoid an excessive increase in the viscosity of the matrix that could compromise the impregnation of the reinforcing fabrics, the lowest temperature explored (200 °C) was chosen for the laminate production. Results from SEC (Table 3) appear to be in line with the rheological characterization. Compared to the initial PA11 pellet, the composite samples showed higher M_n_ and M_w_ and also a higher M_w_/M_n_. This indicates that in the case of flax-based samples, there is an increase in the molecular weight, especially on the high-M_w_ end, while hybrid samples displayed an enlargement on both sides of molecular weight. The very high M_w_ of flax-based composites might come from some branching reactions due to process conditions.

Branching can be caused by oxidation; therefore, the higher fiber areal weight of hybrid fabric might reduce the permeability of air/oxygen during processing which, coupled with lower thickness and higher thermal conductivity, resulted in a shorter cooling time, so the residence time at high temperature was reduced. This would explain the higher degree of branching observed in flax-based laminates.

### 3.2. Thermal and Structural Characterization

#### 3.2.1. Thermogravimetric Analysis (TGA)

**Figure 2:**
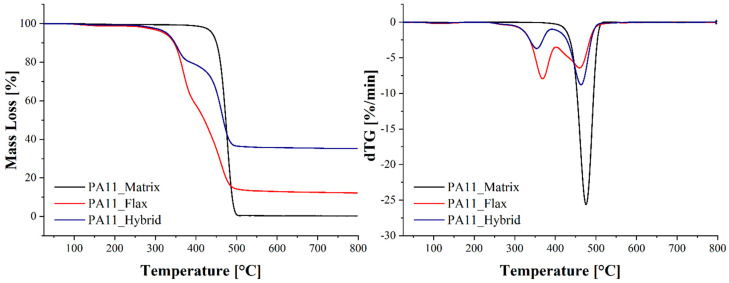
Mass loss and derivative curves of the PA11 matrix and of the flax and flax/basalt hybrid composites.

The thermal stability of the PA11 matrix and the resulting composites, i.e., flax and hybrid, was studied by TGA. The resulting mass loss and derivative curves are shown in Figure 2, while the mass drops and the related degradation temperatures are summarized in Table 4. The neat PA11 matrix is characterized by a single degradation step, with a maximum degradation rate at around 475 °C, while the flax and hybrid composites are characterized by two degradation steps related to flax and PA11 matrix degradation, respectively. The mass loss connected with flax fibers’ degradation is 42.6% for pure flax composite and 22% for hybrid composites and takes place at 368 °C and 354 °C, respectively. These results are in perfect agreement with the ones provided by Lafranche et al. [36], who reported flax degradation at 365 °C, and by Kannan et al. [37], who identified flax degradation in the 333–375 °C temperature range. 

#### 3.2.2. Differential Scanning Calorimetry (DSC) and X-ray Diffraction (XRD)

**Figure 3:**
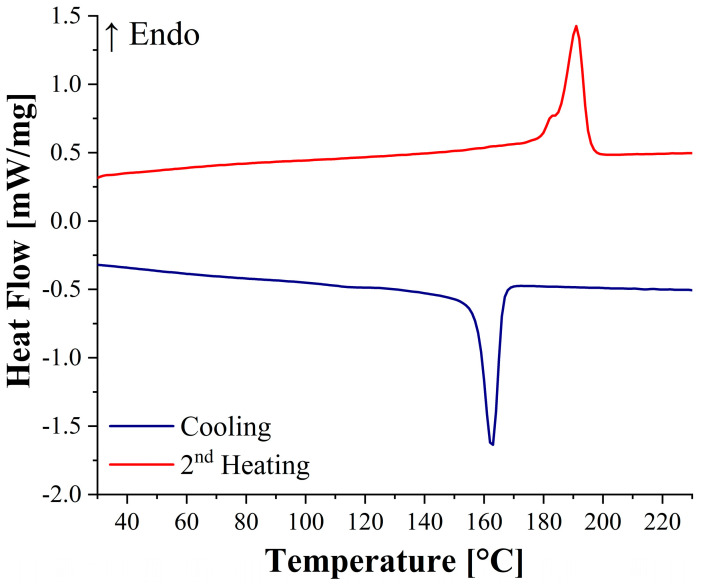
DSC curves of the low molecular weight PA11 matrix.

DSC analysis provided further proof of the 200 °C eligibility as a manufacturing temperature. From the DSC curves of the low molecular weight PA11 shown in Figure 3, it was possible to evaluate the crystallization and melting temperatures of the matrix. In particular, a crystallization temperature of 162.6 °C and a double melting peak at 182.3 °C and 191 °C were detected. This means that 200 °C is the best compromise to achieve full polymer melting while preventing polymer structural changes and deterioration over time. 

A crystallinity degree of 30.3% was calculated, and it is slightly higher than the 27% reported by Russo et al. for a regular PA11 matrix by Arkema [31]. This can be ascribed to the lower molecular weight of the matrix, which ensures an easier macromolecular mobility, thus promoting matrix crystallization [38].

**Figure 4:**
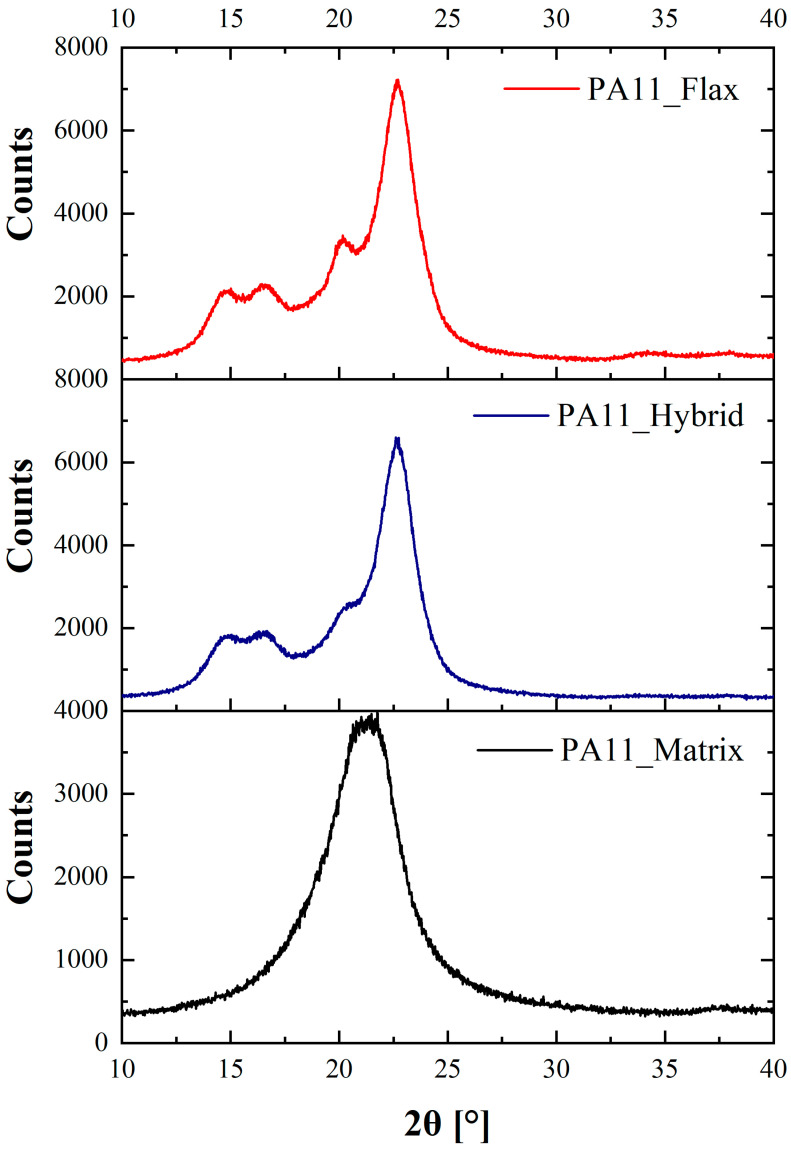
XRD spectra of the as-received PA11_flax, PA11_hybrid composites and PA11_matrix.

As previously mentioned, the PA11 matrix is characterized by a double melting peak ascribable to a two-phase transition resulting from two different crystalline phases [39]. An XRD analysis of the flax and hybrid composites was carried out to confirm this hypothesis, and the resulting XRD spectra are reported in Figure 4. Both flax and hybrid composites display two diffraction peaks at 20.2°–22.7 ° and 20.8°–22.6°, respectively.

The peaks at around 20° can be ascribed to the (200) plane of the triclinic α structure [40] and to the (100) plane of the α’ structure, i.e., a pseudohexagonal δ phase [41]. The peak at around 23° must be ascribed to the (010) and (210) of the triclinic α structure [40]. These results are coherent with the ones reported by Sergi et al. [32] for a regular PA11 matrix. The observation of the two peaks in the XRD spectra resulting from the two different crystalline phases is a valid support to explain the two-stage melting detected in the DSC. Two more peaks at 15.0° and 16.4° for flax composites and at 15.0° and 16.7° for hybrid composites were also identified and must be ascribed to the crystalline component of cellulose I contained in the flax fibers [42]. Indeed, cellulose I also presents a diffraction peak at around 22°–22.8°, which, added to the diffraction resulting from PA11, explains the strong intensity detected at that diffraction angle.

#### 3.2.3. Dynamic Mechanical Analysis (DMA)

**Figure 5:**
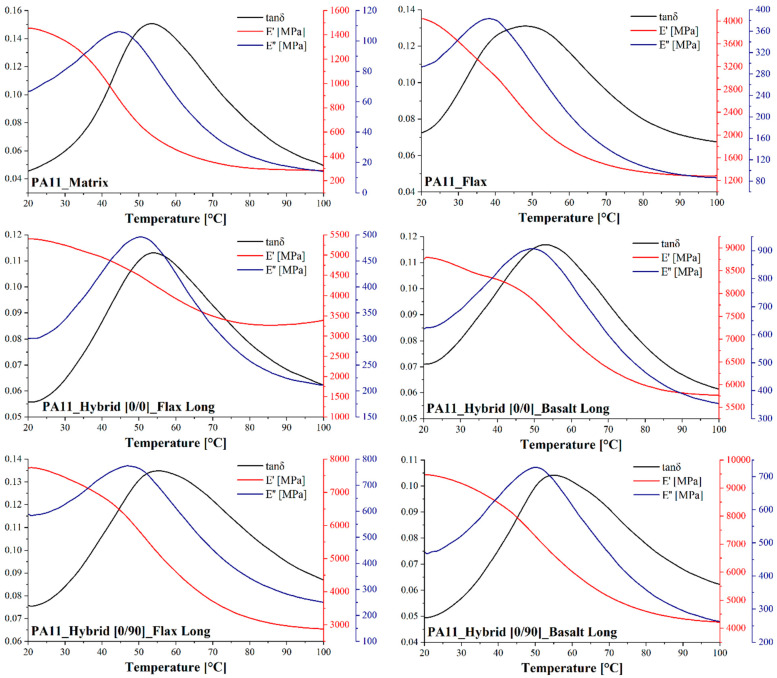
DMA curves of PA11 matrix, flax composite and hybrid [0/0] composites tested along flax and basalt direction and hybrid [0/90] composites tested along flax and basalt direction.

DMA allowed us to evaluate the glass transition temperature and the evolution of storage modulus as a function of temperature for the neat PA11 matrix and for both flax and hybrid configurations accounting also for fiber orientation. Figure 5 shows the DMA curves of PA11 matrix, flax composite and hybrid [0/0] composites tested along the flax and basalt direction and hybrid [0/90] composites tested along the flax and basalt direction, while Table 5 summarizes the glass transition temperature values evaluated from tanδ and the values of E’ at 25, 45, 60 and 80 °C.

All hybrid configurations are characterized by a glass transition temperature comparable with the neat PA11 matrix one at around 53–55 °C. On the contrary, flax laminates exhibit a decrease of 6 °C in this value, probably as a consequence of the plasticizing effect played by moisture, which is absorbed at a larger extent in the composite because of flax fibers’ higher content.

### 3.3. Mechanical Characterization

#### 3.3.1. Flexural Characterization

The quasi-static performance of the bio-based composites under study was evaluated in three-point bending, also assessing the effect of fiber orientation for hybrid configurations. The flexural modulus and strength values are reported in Table 6. The introduction of flax fibers in the PA11 matrix provides an increase of 195% in the bending stiffness and of 78% in the flexural strength, but a further improvement between 380 and 464% in the stiffness and between 112 and 280% in the strength can be achieved by exploiting the hybrid configurations. As expected, flax laminates are characterized by lower flexural properties than hybrids because of the inherent superior mechanical performance of basalt, thus proving that hybridization is a viable solution to produce components with satisfying mechanical requirements while maintaining their partial biodegradability and lightness.

Among hybrids, the [0/0] configuration is characterized by the highest and the lowest flexural stiffness when tested along basalt and flax directions, respectively. Again, the intrinsic higher mechanical performance of basalt fibers ensures the best outcomes when the laminate is tested along their directions, determining a decrease of 15% in stiffness and of 36.6% in strength when tested transversally to their orientation. These results are coherent with the E’ evolution as a function of temperature reported in Table 5. In fact, the [0/0] configuration tested along the basalt direction shows the highest values along the whole temperature range, while the same configuration tested along the flax direction is characterized by the lowest ones. The [0/90] configuration featured intermediate quasi-static properties with a flexural stiffness closer to the [0/0] upper limit. The obtained results are promising, considering that Vitiello et al. [43] reported a 15.7 GPa flexural stiffness and a 174 MPa flexural strength for a regular PA11 matrix reinforced with a basalt 2 × 2 twill, and Vitiello et al. [44] acknowledged a bending stiffness of around 13 GPa and a strength of around 130 MPa for a PA11 reinforced with a plain-weave basalt fabric. The results compare favorably even with more traditional composite configurations, such as polypropylene (PP) glass-reinforced ones. In particular, Russo et al. [45] and Simeoli et al. [46] report a flexural modulus between 15.1 and 15.7 GPa and a flexural strength between 112 and 183 MPa working with fiber volume fractions around 0.5 and 0.54, which are higher than the ones reported in this work, respectively. 

**Figure 6:**
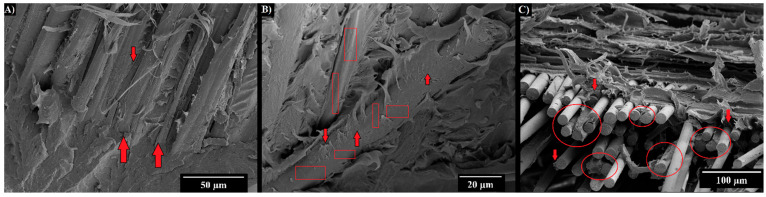
SEM micrographs detailing the fracture surface of flax (**A**,**B**) and hybrid composites (**C**).

Tested specimens were further analyzed through SEM to investigate composite fracture surface, and Figure 6 shows the fracture surface of both flax (A) and hybrid (B and C) laminates. Figure 6A,B highlight some interfacial adhesion issues arising between the extremely hydrophilic flax and the PA11 matrix. In particular, Figure 6A shows a partial detachment of the matrix from the fibers, proving that further improvements can be achieved to increase matrix/fiber load transfer. Figure 6B, in turn, underlines that PA11/flax compatibility is better compared to a more hydrophobic matrix such as polypropylene and polyethylene, but there is certainly further room for improvement. In particular, the flax fiber displays a surface with some residual traces of matrix proving some degree of compatibility. Better results in terms of compatibility were achieved between PA11 and basalt fibers, as shown in Figure 6C. Despite the presence of fiber pull out, basalt fibers display many residual traces of PA11 on their surface, which prove to successfully transfer the load from the matrix to the fibers. If the improvement of the quasi-static properties of the proposed bio-based composites is the main goal from the design perspective, many physical and chemical treatments can be exploited to improve flax and PA11 adhesion and the resulting load bearing capabilities. Quiles-Carrillo et al. [47] demonstrated that the introduction of glycidyl-silane and amino-silane in bio-PA10-10/slate composites allows for the increase in their tensile strength and stiffness. Moreover, a good interfacial adhesion was achieved by Armioun et al. [30] in PA11/wood fiber composites by adding a maleic anhydride grafted polypropylene as coupling agent.

#### 3.3.2. Impact Characterization

**Figure 7:**
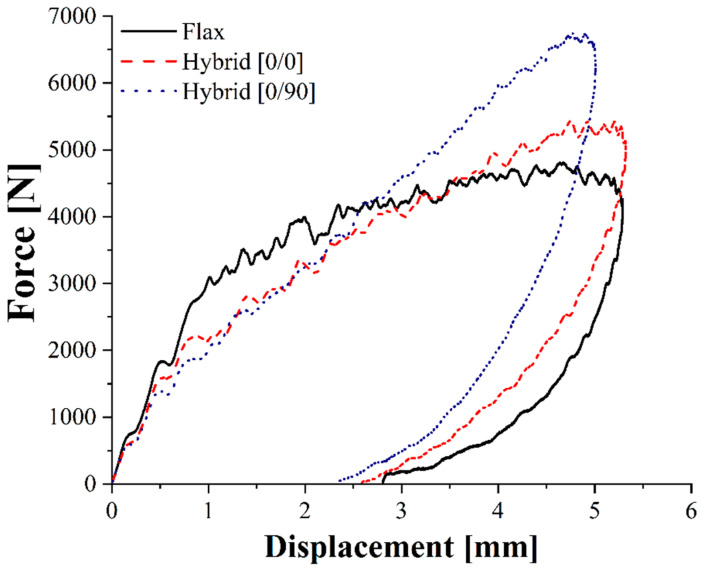
Force-displacement curves of flax and hybrid composites for a 20 J impact at room temperature.

Quasi-static mechanical properties are important to ensure composites use, but their impact response is also crucial to ensure their applicability in the transportation sector. The main impact properties of flax, [0/0] and [0/90] composites for different impact energies at room temperature are summarized in Table 7, while Figure 7 shows the force-displacement curves of the aforementioned laminates impacted at 20 J.

**Figure 8:**
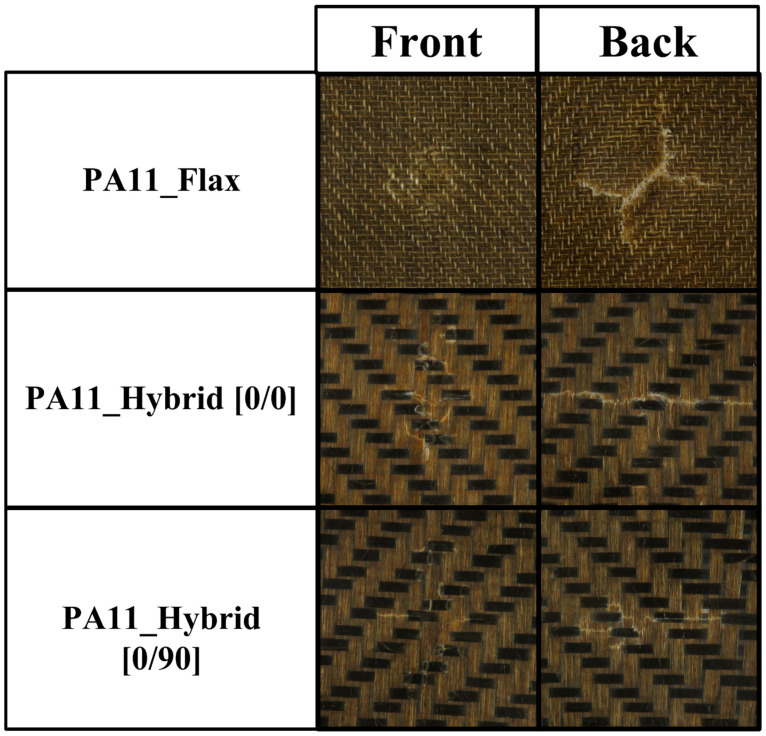
Front and back damage mode of flax, hybrid [0/0] and hybrid [0/90] laminates impacted at room temperature at 20 J.

**Figure 9:**
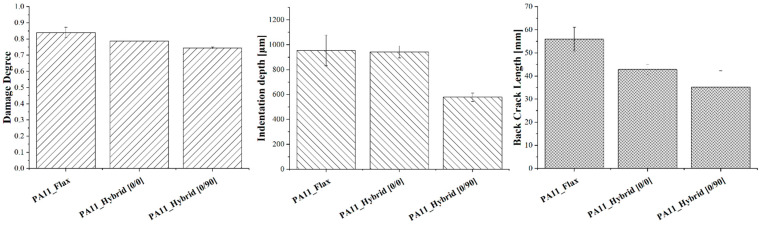
Damage degree, permanent indentation and back crack extent of flax and hybrid laminates after a 20 J impact at room temperature.

As already acknowledged in flexural tests, both hybrid configurations considerably outperform flax–PA11 composites displaying a higher maximum force, a lower maximum displacement and therefore a higher stiffness. Moreover, flax composites are always characterized by a higher damage degree, defined as the ratio of absorbed to impact energy, which can compromise their integrity and their residual mechanical properties. The higher damage extent is also confirmed by laminates’ damage mode shown in Figure 8 and permanent indentation and back crack extent data summarized in Figure 9 for a 20 J impact.

Concerning biocomposite damage mode, hybrid configurations display a single crack on the rear side which runs perpendicularly to flax fibers due to the lower strength of these fibers, which act as a damage preferential path requiring a lower energy to propagate. On the contrary, PA11–flax composites are characterized by a cross-shaped rear crack which derives from their symmetric nature and the circularity of the sample holder where no preferential paths occur. Focusing on the damage extent, flax composites display a much higher back crack extent than hybrids and a higher permanent indentation than hybrid [0/90], thus confirming that the higher amount of energy absorbed, and the higher damage degree, can significantly jeopardize their residual structural performance.

Among the two hybrid configurations, [0/90] laminates respond better to the impact than [0/0] ones exhibiting the highest maximum force and the lowest maximum displacement and damage degree. This must be attributed to the lower anisotropy of the [0/90] configuration resulting from fabric orientation alternation which involves basalt fibers orientation along two different directions. This ensures satisfying load-bearing capabilities along two perpendicular directions rather than one, thus delaying flax fibers’ overloading and breakage. This is confirmed by the damage-extent analysis. In fact, [0/90] hybrids are characterized by a decrease of 38.6% in the permanent indentation and of 17.7% in back crack extent.

**Figure 10:**
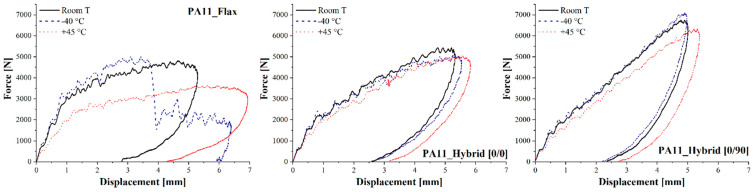
Force-displacement curves of flax and hybrid composites for a 20 J impact at −40 °C, room temperature and +45 °C.

The effect of low and high temperatures on the impact response of the bio-based composites under study was assessed, and Figure 10 shows the impact response curves, i.e., force against displacement, of flax, [0/0] and [0/90] hybrid laminates impacted at 20 J at −40 °C, room temperature and +45 °C. Regardless of the operating temperature, flax-reinforced PA11 is always characterized by the worst impact response displaying the lowest maximum force and the highest maximum displacement.

For all composite configurations, the progressive increase in operating temperature induces a decrease in laminates’ maximum force and an increase in the maximum displacement, which derives from a progressive decrease in laminates’ stiffness. This effect becomes particularly evident at +45 °C, which is extremely close to the PA11 matrix glass transition temperature, and a transition from the glassy to the rubbery state can significantly modify composite response. The stiffer, even if more brittle, response of the laminates for decreasing operating temperature is also confirmed by flax composites, which undergo significant penetration phenomena when impacted at 20 J and −40 °C while remaining in the elastic rebound region when tested at room temperature and +45 °C.

**Figure 11:**
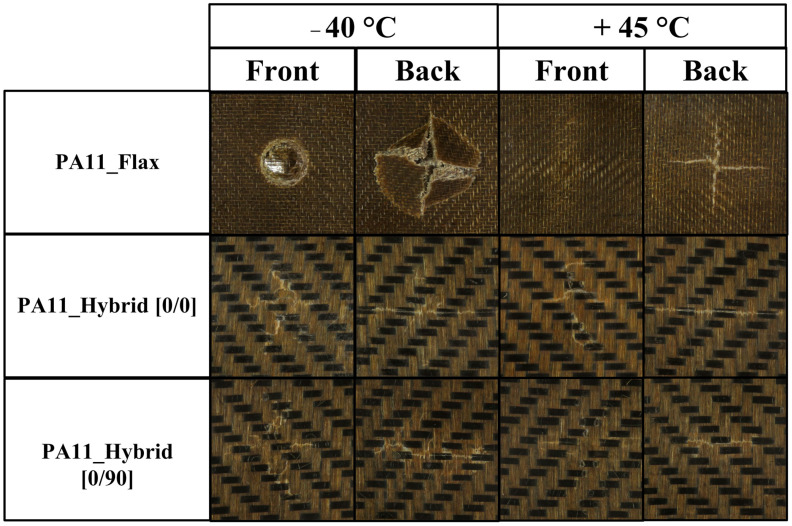
Front and back damage mode of flax, hybrid [0/0] and hybrid [0/90] laminates impacted at 20 J at −40 °C and +45 °C.

**Figure 12:**
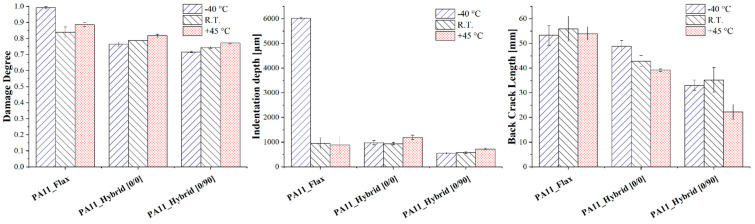
Damage degree, permanent indentation and back crack extent of flax and hybrid laminates after a 20 J impact at −40 °C, room temperature and +45 °C.

The progressive approach of matrix glass transition temperature determines its progressive softening, and the resulting reduction in stiffness makes the laminate more compliant toward the impact, allowing it to involve a wider area of the specimen, thus preventing damage localization and penetration phenomena. All these considerations are supported by the composite damage mode shown in Figure 11 and the damage extent analysis reported in Figure 12. Considering flax composites, the highest damage degree is achieved at −40 °C due to the brittle response of the laminate, which leads to a strong penetration of the impactor and appears in the form of a pronounced permanent indentation, i.e., six times higher than the one of specimens impacted at room temperature and +45 °C.

Different is the trend for hybrid laminates, where a progressive increase in damage degree can be observed for increasing operating temperatures while keeping the permanent indentation almost constant and decreasing the back crack extent. Furthermore, hybrids impacted at −40 °C display some brittle cracks arising on the front side of the specimen near the contact area with the impactor. These last items become progressively less branched and pronounced when working at higher operating temperatures. This can be explained considering that the laminates are approaching PA11 glass transition temperature, thus making the matrix more prone to plastic deformation and activating further energy dissipation mechanisms. This allows for an increase in the amount of energy that the laminate is able to store, keeping constant the local indentation and delaying the formation of the back crack resulting from the approach of the bending elastic limit of the laminate.

## 4. Conclusions

The present research work investigated the thermal, quas-istatic and impact properties of a low molecular weight bio-based PA11 reinforced with flax and intraply flax/basalt hybrid fabrics to assess the feasibility of these bio-based composites for the automotive sector. For hybrid composites, the effect of stacking sequence, i.e., [0/0] and [0/90], and fiber orientation were considered, while the effect of temperature, i.e., −40 °C, room temperature and +45 °C, was investigated for laminates’ impact properties. The main outcomes of the study are as follows:

- The low molecular weight of the PA11 matrix ensures an easier manufacturing of the composites displaying a good processability already at 200 °C. This allows one to obtain components of good quality while preventing matrix and, above all, flax fibers’ thermal degradation.
- Both quasi static and impact tests proved that hybrid laminates significantly outperform flax ones, thus proving that hybridization is an effective way to improve the mechanical performance of bio-based composites while preserving their lightness.
- Among the hybrid configurations under study, the [0/90] is the best at ensuring a satisfying bending stiffness and an improved impact resistance deriving from the higher isotropy in basalt fibers’ orientation, which allows for a better distribution of the impact load and delays flax fibers’ overloading and breakage.
- Operating temperature strongly influences composite impact properties, determining a progressive decrease in matrix, and therefore in laminate, stiffness, which makes the component more compliant toward the impact, thus involving a wider area of the specimen and preventing damage localization.

As already discussed, the results obtained for the [0/90] configuration compare favorably with PA11 fully basalt-reinforced composites, but also with more traditional composite configurations such as PP/glass-reinforced ones, thus validating the viability of this bio-based solution for the manufacturing of more eco-friendly and sustainable automotive components. In addition, this material combination might provide a better recyclability compared to thermoset-based composites, as PA11/flax composites have already proven to be very efficient after recycling [48], a behavior that can be even improved by the presence of basalt fibers.

## Figures and Tables

**Table 1:** Main characteristics of the three composite configurations under study.

Composite	Reinforcement Type	Stacking Sequence	Thickness (mm)	Fiber Volume Fraction
PA11_Flax	Flax	[0/0]_s_	5.30 ± 0.05	0.40
PA11_Hybrid [0/0]_s_	50% Flax/50% Basalt	[0/0]_s_	4.40 ± 0.05	0.44
PA11_Hybrid [0/90]_s_	50% Flax/50% Basalt	[0/90]_s_	3.90 ± 0.05	0.49

**Table 2:** Impact energy and temperature combinations investigated for each PA11 composite configuration.

Laminate	Flax	Hybrid [0/0]_s_	Hybrid [0/90]_s_
−40 °C	10 J, 20 J	10 J, 20 J, 30 J	20 J
Room Temperature	10 J, 20 J, 30 J	10 J, 20 J, 30 J	20 J
+45 °C	10 J, 20 J, 30 J	10 J, 20 J, 30 J	20 J

**Table 3:** Molecular weights obtained by size-exclusion chromatography.

Composite	M_n_ (g/mol)	M_w_ (g/mol)	M_z_ (g/mol)	M_w_/M_n_	M_z_/M_w_
PA11_Matrix	25,900	47,200	69,900	1.8	1.5
PA11_Flax	30,600	109,900	497,600	3.6	4.5
PA11_Hybrid	23,400	54,400	309,900	2.3	5.7

**Table 4:** Mass drops and related degradation temperatures for the PA11 matrix and the flax and flax/basalt hybrid composites.

	PA11_Matrix	PA11_Flax	PA11_Hybrid
1st drop temperature (°C)	-	368.5	354.0
1st mass loss (%)	-	42.6	22.0
2nd drop temperature (°C)	475.6	459.6	463.3
2nd mass loss (%)	100	44.5	42.7

**Table 5:** Glass transition temperature and E’ evolution as a function of temperature of PA11 matrix, flax composite and hybrid [0/0] composites tested along flax and basalt direction and hybrid [0/90] composites tested along flax and basalt direction.

	Glass Transition Temperature [°C]	E’(25 °C) [MPa]	E’(45 °C) [MPa]	E’(60 °C) [MPa]	E’(80 °C) [MPa]
PA11_Matrix	53.4	1420.9	847.0	454.9	303.8
PA11_Flax	48.0	3899.6	2650.5	1748.3	1353.0
PA11_Hybrid [0/0]_Basalt_Long	52.9	8730.4	8125.4	7006.6	5981.5
PA11_Hybrid [0/0]_Flax_Long	53.9	5353.2	4739.3	3924.8	3296.2
PA11_Hybrid [0/90]_Basalt_Long	55.2	9378.0	7951.6	6012.8	4603.3
PA11_Hybrid [0/90]_Flax_Long	55.0	7657.4	6437.0	4590.8	3204.2

**Table 6:** Flexural modulus and strength values for flax and hybrid configurations along flax and basalt directions.

	Flexural Modulus [GPa]	Flexural Strength [MPa]
PA11_Matrix	1.74 ± 0.14	72.33 ± 3.35
PA11_Flax	5.13 ± 0.46	128.47 ± 6.34
PA11_Hybrid [0/0]_Basalt_Long	9.82 ± 0.36	276.14 ± 9.38
PA11_Hybrid [0/0]_Flax_Long	8.34 ± 0.29	175.03 ± 5.95
PA11_Hybrid [0/90]_Basalt_Long	9.4 ± 0.31	153.69 ± 7.27
PA11_Hybrid [0/90]_Flax_Long	9.79 ± 0.20	218.55 ± 6.33

**Table 7:** Main impact properties, i.e., maximum force, maximum displacement and damage degree, of flax, [0/0] and [0/90] hybrid composites at room temperature at different impact energies.

		PA11_Flax	PA11_Hybrid [0/0]	PA11_Hybrid [0/90]
**10 J**	Max Force [N]	4244.2 ± 89.20	4200.1 ± 0.70	-
	Max Displacement [mm]	3.25 ± 0.09	3.55 ± 0.02	-
	Damage Degree	0.76 ± 0.03	0.74 ± 0.01	-
**20 J**	Max Force [N]	4879.8 ± 97.70	5342.2 ± 148.60	6793.1 ± 61.00
	Max Displacement [mm]	5.26 ± 0.05	5.38 ± 0.08	5.01 ± 0.01
	Damage Degree	0.84 ± 0.03	0.79 ± 0.01	0.74 ± 0.01
**30 J**	Max Force [N]	5179.1 ± 70.00	5614.8 ± 18.20	-
	Max Displacement [mm]	12.36 ± 0.04	7.43 ± 0.02	-
	Damage Degree	1.00 ± 0.01	0.84 ± 0.01	-

## Data Availability

All data are contained within the article.
